# Significance of sphingosine kinase 1 expression in feline mammary tumors

**DOI:** 10.1186/s12917-019-1883-z

**Published:** 2019-05-17

**Authors:** Yi-Chih Chang, Hsiao-Li Chuang, Ji-Hang Yin, Jiunn-Wang Liao, Ter-Hsin Chen, Yu-Chih Wang

**Affiliations:** 1 Department of Biotechnology, College of Medical and Health Science, Asia University, Taichung, Taiwan; 2 Department of Medical Laboratory Science and Biotechnology, China Medical University, Taichung, Taiwan; 3 National Laboratory Animal Center, National Applied Research Laboratories, Taipei, Taiwan; 4 Graduate Institute of Veterinary Pathobiology, National Chung Hsing University, Yu-Chih Wang, 145 Xingda Rd., South Dist, Taichung City, 402 Taiwan

**Keywords:** Feline mammary tumor, SPHK1 - comparative oncology

## Abstract

**Background:**

Sphingosine kinase 1 (SPHK1) is an enzyme that converts pro-apoptotic ceramide and sphingosine into anti-apoptotic sphingosine-1-phosphate. There is growing evidence that SPHK1 activation promotes oncogenic transformation, tumor growth, chemotherapy resistance, and metastatic spread. High SPHK1 expression has been associated with a poor prognosis in several human cancers.

**Results:**

In the present study, the expression level of SPHK1 was examined in feline mammary tumor (FMT) specimens, and the IHC expression level of SPHK1 was associated with the histological grade of FMTs. IHC analysis of 88 FMT cases revealed that the expression level of SPHK1 was upregulated in 53 tumor tissues (60.2%) compared to adjacent mammary tissues. SPHK1 expression in FMTs was significantly associated with histological grade, presence of lymphovascular invasion, and estrogen receptor negativity. Treatment of primary FMT cells with SPHK1 inhibitors reduced cell viability, indicating that SPHK1 acts to promote FMT cell survival. These results indicate that SPHK1 may play an important role in FMTs and may be a therapeutic target in cats with FMT.

**Conclusions:**

SPHK1 over-expression in breast cancer tissues is associated with a poor prognosis in humans. SPHK1 over-expression in more aggressive FMTs provides support for a potential role of SPHK1 inhibitors for the treatment of FMTs. Targeting SPHK1 has potent cytotoxic effects in primary FMT cells. These findings suggest that further examination of the role SPHK1 plays in FMTs will pave the way for the investigation of SPHK1 inhibitors in future clinical applications.

**Electronic supplementary material:**

The online version of this article (10.1186/s12917-019-1883-z) contains supplementary material, which is available to authorized users.

## Background

Feline mammary tumors (FMT) are the third most common type of neoplasm found in female cats [1]. FMT tend to be malignant (malignancy rates range between 80 and 90%) [2, 3]. The overall survival time can vary significantly depending on the tumor size [1, 4–6], World Health Organization stage [5, 6], histologic grade [1], tumor subtype [1], nuclear pleomorphism [1] and lymphovascular invasion [1, 5, 6]. Increased expression of vascular endothelial growth factor, estrogen receptor (ER), progesterone receptor, AKT [7], epidermal growth factor receptor 2 (HER-2) [8, 9], cyclooxygenase-2 [10, 11], Ki-67 [5, 12, 13] and p53 mutations [14, 15] have been investigated to identify prognostic molecular markers of FMTs, but only a few markers have entered clinical use. Therefore, a more appropriate therapeutic target is needed to improve the understanding and biological characterization of FMTs and therapeutic development.

Sphingosine kinase 1 (SPHK1) is a rate-limiting enzyme that functions to phosphorylate sphingosine to form sphingosine-1-phosphate (S1P). The SPHK1/S1P-signaling axis regulates important cellular and pathophysiological functions. Growing evidence shows that tumor cells upregulate SPHK1 expression to release more S1P into the tumor microenvironment [16]. The increased S1P acts in an autocrine or paracrine manner on tumor cells to inhibit cell apoptosis, promote survival signals, promote angiogenesis, and enhance the metastatic potential of tumor cells [16–18]. Therefore, SPHK1/S1P signaling could be a useful target for cancer therapy.

Upregulation of SPHK1 has been reported to have the potential to act as a prognostic biomarker in many human cancers, including oral squamous cell carcinoma [19], prostate cancer [20], gastric cancer [21], astrocytoma [22], and breast cancer [23, 24]. However, investigation of SPHK1 expression in feline tumors is limited. In this study, we examined SPHK1 protein expression levels in primary FMT cells using tissue micro-array (TMA) and immunohistochemical (IHC) staining. In addition, we investigated the association between SPHK1 expression and the histopathological characteristics of FMT.

## Results

### Histopathological features evaluation

Histopathological results of the 88 FMT cases are shown in Table 1, and the histological features were identified including malignant features such as carcinoma with a simple tubular or tubulopapillary type, solid type, and comedocarcinoma. There were 2 normal mammary tissues and 5 non-neoplastic lesions, including normal lobular hyperplasia and duct ectasia. Feline mammary carcinomas were classified according to the malignancy criteria established by Elston and Ellis [25] as grade 1 (57 cases, 64.7%), grade 2 (24 cases, 27.3%), and grade 3 (7 cases, 8.0%) or by Mills et al. [1] as grade 1 (43 cases, 48.9%), grade 2 (30 cases, 34.1%), and grade 3 (15 cases, 17.0%) (Table 1). The status of ER and the expression of feline HER2 were evaluated in all FMTs. Fourteen cases (15.9%) were ER positive and 45 cases (51.1%) were classified as positive for HER2 expression.

**Table 1:** Demographics of feline mammary cases

	Malignant tumor				
	Tubular	Tubulopapillary	Solid	Comedo	Total
Histological grade (Elston and Ellis)					
1	36	4	15	2	57
2	15	0	8	1	24
3	2	0	3	2	7
Histological grade (Mills et al.)					
1	29	2	11	1	43
2	21	1	8	0	30
3	3	1	7	4	15
ER-positive	11	1	2	0	14
HER2-positive	28	1	13	3	45
SPHK1-positive	29	2	17	5	53

### Upregulated SPHK1 expression in feline mammary tumors

**Fig. 1:**
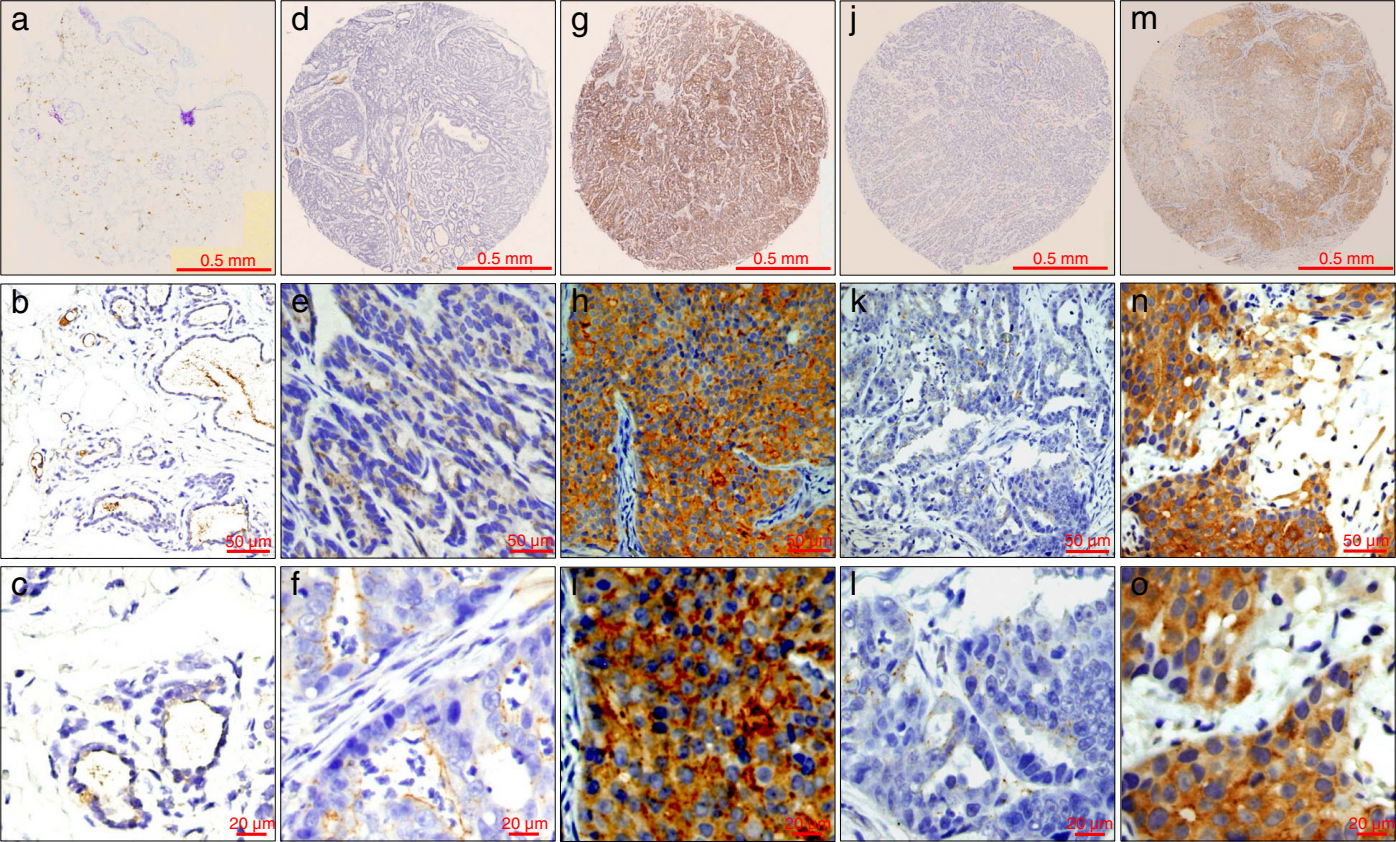
Immunohistochemical staining of SPHK1 was carried out in FMT TMA tissues. **a-c** Low SPHK1 expression in normal mammary tissues. **d-f** Low SPHK1 expression in FMT. **g-i** Upregulated SPHK1 expression in FMT. **j-l** Negative control (rabbit antibody against SPHK1 was omitted, but normal rabbit IgG was applied). **m-o** Positive control (xenografted human breast cancer MCF-7 cells)

**Fig. 2:**
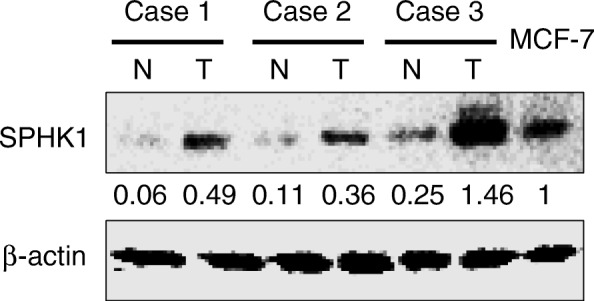
SPHK1 expression levels in 3 paired FMT (T) and adjacent normal tissues (N). MCF-7 cells as positive control. β–actin as loading control. Relative quantitation to MCF-7 cells and normalization to β–actin was calculated by using ImageJ

Comparative studies showed that FMTs have much similarities to human mammary tumors [8, 26]. We were interested in determining whether SPHK1 is dysregulated in FMTs, which are similar to human breast cancers. In IHC analysis, there was negative or weak staining in normal and adjacent non-neoplastic mammary tissues (Fig. 1a-f). Positive cytoplasmic and membranous staining for SPHK1 could be observed in FMTs (Fig. 1g-i). Expression of SPHK1 with molecular weight between 40 and 50 kDa was found in both the non-neoplastic and the FMT tissues. Western blot analysis showed an increase in the average signal intensity of SPHK1 in FMT tissues compared with non-neoplastic tissues (Fig. 2).

**Fig. 3:**
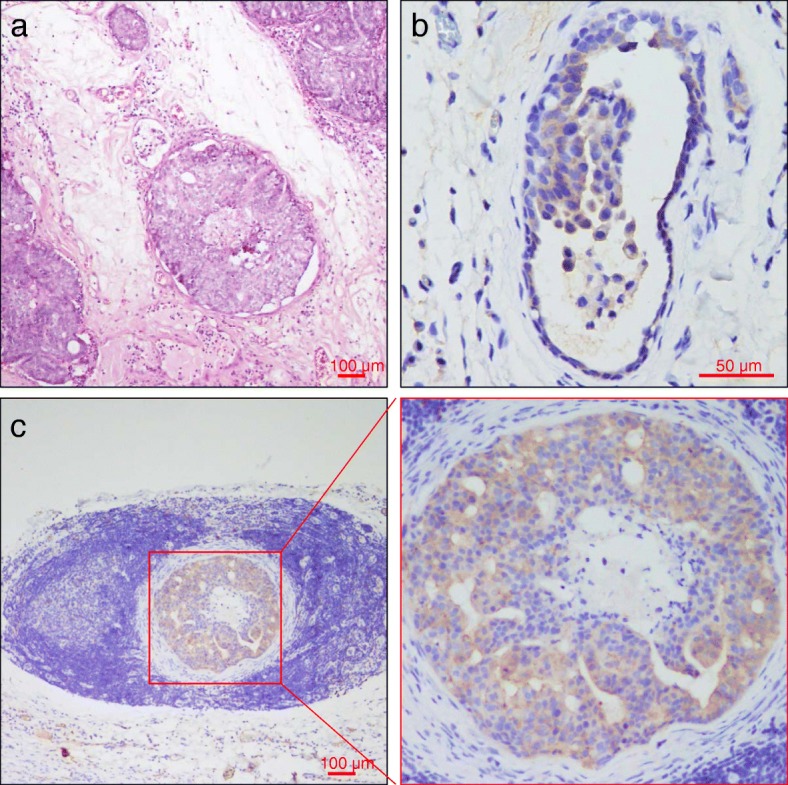
Representative microphotograph of lymphovascular invasion in a FMT. **a** Hematoxylin-Eosin stained microscopy image of tumor lymphovascular emboli. SPHK1-positive tumor emboli were observed in lymphovascular vessel (**b**) and lymph node (**c**)

Higher expression of SPHK1 [Additional file 2] in carcinomas was found to be associated with FMT malignancy (histologic grading by Elston and Ellis/ Mills et al. systems) (Table 2, Additional file 3). Although there was no statistical difference, SPHK1 expression levels in FMTs tended to be higher in patients with low tubule formation, more nuclear pleomorphism or lymphocytic infiltration. There was no statistically significant association between SPHK1 expression and mitotic count, ulceration, and necrosis (Table 2). SPHK1-positive FMT cells were found in lymphovascular invasion and lymph node metastasis FMT tissues (Fig. 3). We further investigated the association between levels of SPHK1 in FMT tissue and ER and HER2 expression status. High SPHK1 expression was found in 2 of the ER-positive (2/14, 14.3%) and 24 of the HER2-positive (24/45, 53.3%) FMT tissues. Significant correlation was observed between high SPHK1 expression and ER negativity (*P* = 0.009). There was no significant association between high SPHK1 expression and HER2 expression (*P* = 0.325; Table 3).

**Table 2:** Relationship between SPHK1 expression and histopathological factors

	SPHK1 positive	Lower 95% CI	Upper 95% CI	*p*-value
Histologic grade (Elston and Ellis)				
Grade 1	50.88%	37.29%	64.37%	0.024
Grade 2	79.17%	57.85%	92.87%	
Grade 3	71.43%	29.04%	96.33%	
Histologic grade (Mills et al.)				0.0009
Grade 1	41.86%	27.01%	57.87%	
Grade 2	73.33%	54.11%	87.72%	
Grade 3	86.67%	59.54%	98.34%	
Tubule formation				0.112
< 10%	47.83%	26.82%	69.41%	
10–75%	69.23%	52.43%	82.98%	
> 75%	57.69%	36.92%	76.65%	
Nuclear pleomorphism				0.099
< 5% abnormal	48.98%	34.42%	63.66%	
> 5% abnormal	74.36%	57.87%	86.96%	
Mitotic count				0.457
< 62	58.75%	47.18%	69.65%	
> 62	75.00%	34.91%	96.81%	
Lymphovascular invasion				0.016
Absent	49.23%	36.60%	61.93%	
Present	91.30%	71.96%	98.93%	
Lymphocytic infiltration				0.112
Absent	70.59%	56.17%	82.51%	
Present	45.95%	29.41%	63.01%	
Ulceration				0.433
Absent	42.86%	57.14%	9.9%	
Present	61.73%	38.27%	50.26%	
Necrosis				0.429
Absent	52%	48%	31.31%	
Present	63.49%	36.51%	50.4%

**Table 3:** Relationship between SPHK1 expression and ER / HER2 status

SPHK1 positive	Lower 95% CI	Upper 95% CI	*p*-value	
ER				
Negative	68.92%	57.10%	79.17%	0.009
Positive	14.29%	1.78%	42.81%	
HER2				
Low	67.44%	51.46%	80.92%	0.325
High	53.33%	37.87%	68.34%	

### Inhibition of SPHK1 activity induces apoptosis and inhibits proliferation of FMT cells

**Fig. 4:**
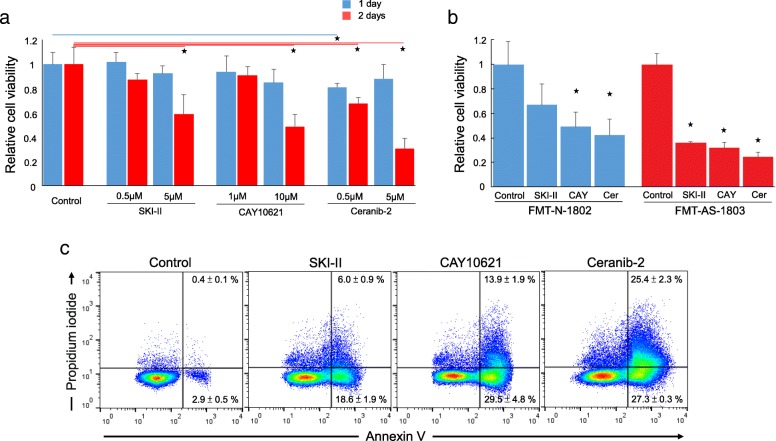
Effect of SKI-II, CAY10621 and Ceranib 2 on the cell viability determined by WST assay. **a** FMT-CH-1801 cells were treated with SKI-II, CAY10621 or Ceranib 2 at the indicated concentration for 24 (blue) and 48 (red) hours. Bar represents mean ± SD. *p* value < 0.05. **b** FMT-N-1802 (blue) and FMT-AS-1803 (red) cells treated with SKI-II, CAY10621 or Ceranib 2 for 2 days. Bar represents mean ± SD. *p* value < 0.05. **c** Annexin-FITC and PI staining after FMT-N-1802 cells treated with SKI-II, CAY10621 or Ceranib 2 for 2 days. The Annexin-FITC positive and PI negative cells represent early apoptotic cells. The Annexin-FITC and PI positive cells represent late apoptotic cells

To test the effect of inhibiting SPHK1 activity on FMT cells, we treated the primary ER-negative FMT cells (FMT-CH-1801) with the SPHK1 inhibitor (SKI-II and CAY10621) or acid ceramidase inhibitor (ceranib-2). The three inhibitors targeting SPHK1 showed cytotoxic effect against FMT cells isolated from primary FMT tissue. The results of the cell viability assay indicated that 5 μM SKI-II, 10 μM CAY10621 or 5 μM ceranib-2 significantly reduced FMT-CH-1801 cell viability after 48 h of treatment (Fig. 4a). The cell viability reduction was also observed cells treated with 0.5 μM Ceranib-2. This finding was validated in two other primary ER-negative FMT cell lines (FMT-N-1802 and FMT-AS-1803). SPHK1 inhibitors also showed significant cytotoxicity in the other two FMT cell lines (Fig. 4b). Simultaneous stained with annexin V-FITC and IP to distinguish between intact cells (both annexin V and PI negative), early apoptosis (annexin V-positive and PI-negative), and late apoptosis (both annexin V and PI positive). A significant elevation in the level of early and late apoptotic cells was detected in FMT-N-1802 cells treated with SKI-II, CAY10621 or ceranib-2. The rates of apoptotic cells (early apoptosis + later apoptosis) were rasied to 24.6% (18.6 + 6.0), 43.4% (29.5 + 13.9), and 52.7% (27.3 + 25.4), respectively, compared with the control, which was 3.3% (Fig. 4c).

## Discussion

In the present study, we analyzed SPHK1 expression in FMT tissues. Consistent with previous human breast cancer reports [24, 27], our current study showed that levels of SPHK1 were higher in FMTs compared with normal tissue from the same patient. There was an association between increased SPHK1 expression and aggressive oncogenic behaviors in FMTs, including higher histological grade, lymphovascular invasion, and ER negativity. Both the presence of lymphovascular invasion and the tumor grade are reliable prognostic parameters for FMTs [1, 6]. These findings implicate SPHK1 as a potentially important contributing factor in FMT cancer progression and metastasis. High SPHK1 expression associated with lymphatic metastasis is also found in human breast cancer [28]. Activation of SPHK1 encourages tumor progression by promoting angiogenesis and lymphangiogenesis in human breast cancer cells [17, 27]. Thus, alterations in SPHK1 expression potentially promote tumor development and progression of FMTs, and SPHK1 should be further investigated as a potential biomarker to predict clinical FMT patient outcome.

Since high SPHK1 expression is correlated with ER negativity (Table 3), the different proportions of ER negative FMTs in groups with high and low SPHK1 expression could confound the results. SPHK1 has been linked to estrogen signaling [29] and estrogen-dependent tumorigenesis in MCF-7 cells [25]. The mechanism leading to higher SPHK1 levels in ER-negative FMTs is still unclear. Leptin-mediated SPHK1 expression is found in ER-negative breast cancer cells, but absent in ER-positive breast cancer cells [30], suggesting that the differential activation of leptin-mediated signaling response to ER may further regulate the expression of SPHK1.

## Conclusions

SPHK1 inhibitor or acid ceramidase inhibitor induced cytotoxicity of primary FMT cells points to the possibility of SPHK1 being a molecular target for FMT therapy. Future application of specific SPHK1 inhibitors in xenograft model will aid in elucidating its importance in the progression of FMTs and the usefulness of targeting SPHK1 for FMT therapy.

## Methods

### Tissue samples and histology

FMT tissue samples from 88 domestic cats were collected by the MorningStar animal hospital (Taichung, Taiwan) between 2015 and 2017. Samples were fixed in 4% neutral buffered formalin, paraffin embedded, sectioned at 4 μm and stained with haematoxylin and eosin. Histologic grading was evaluated according to the Elston and Ellis [31] or Mills et al. [1] grading systems, listed in Additional file 1: Table S1. Xenografted human MCF-7 tissue was used as a positive control, and samples stained with rabbit IgG isotype antibody (ThermoFisher, Cat. 02–6102) served as a negative control. A case was considered positive for lymphovascular invasion when neoplastic emboli were found within endothelium-lined vessel lumens (Fig. 3a). In cases with lymphovascular invasion, additional sections were stained with Von Willebrand factor antibody for vascular endothelium.

### Tissue microarray

Tissue microarrays of FMTs were constructed by using an EZ-TMA 72-core manual tissue arrayer (IHCworld). Briefly, on H&E stained slides, representative areas of tumor were marked. The TMA was then constructed from corresponding areas in the paraffin-embedded block of a primary FMT, and cores of 1.5 mm in diameter from each FMT block were arrayed into a recipient block. After array construction, a section was stained with H&E to confirm the presence of neoplastic tissue.

### Immunohistochemistry

The expressions of ER, fHER2 and SPHK1 were evaluated immunohistochemically using the avidin-biotin immunoperoxidase method. TMA sections were deparaffinized by xylene, and rehydrated with graded ethanol. Following blocking of endogenous peroxidase activity with 3% hydrogen peroxide in methanol for 10 min, antigens were retrieved with 10 mM citrate buffer. The anti-ER antibody (clone 6 F11, 1:100 dilution; Thermo Scientific) [9], the anti-HER2 antibody (clone CB11, 1:200 dilution; Invitrogen) [26], or the anti-SPHK1 polyclonal antibody (1:200 dilution; cat. 3297; Cell Signaling Technology) were incubated with TMA sections for 1 h at room temperature. The sections were then incubated with biotinylated secondary immunoglobulin G and then horseradish peroxidase-conjugated streptavidin. Subsequent to washing with phosphate-buffered saline buffer, the antibody complexes were visualized with 3,3′-diaminobenzidine tetrahydrochloride as chromogen and slides were counterstained with Gill’s haematoxylin. Expression from IHC assays was scored by veterinary pathologists (Ji-Hang Yin, Jiunn-Wang Liao and Yu-Chih Wang) blinded to the origination of the samples. Scores were assigned according to the intensity of the cytoplasmic and/or membrane staining and the extent of stained cells. The final score was calculated by multiplying the intensity score as (no staining, 0; weak staining, 1; moderate staining, 2; and strong staining, 3) with the extent of staining score (0%, 0; 1–24%, 1; 25–49%, 2; 50–74%, 3; and 75–100%, 4). A tumor was considered to have high SPHK1 expression when achieving a score ≥ 6.

### Primary FMT cells cultures

Three FMT lesions, collected immediately after surgical removal, were obtained from the Nyan cat clinic, An-Sing animal hospital and the veterinary medical teaching hospital of National Chung-Hsing University. Solid FMT tissue was cut into small pieces using a scalpel or scissors. The tissue was digested into organoids with digestion buffer (2 mg/ml Type 3 collagenase (Worthington) and 100 U/ml hyaluronidase (Sigma) in DMEM medium) in a 200 rpm shaker at 37 °C overnight. The organoids were digested by TrypLE (Gibco) for 10 min at 37 °C. The isolated cells were cultured in DMEM medium containing 10% fetal bovine serum. FMT cells were immunomagnetically deprived with anti-fibroblast microbeads (Miltenyi Biotec) to avoid fibroblast contamination.

### Cell viability assay

Primary FMT cells were seeded into 96-well plates at the concentration of 1 × 10^4^ per well, then cells were treated with doxorubicin or SPHK1 inhibitors for 24 to 48 h. WST assay was used for detecting FMT cell viability. After treatment, 10 μL/well WST reagent was added to the 96-well plates, then incubated at 37 °C, in a 5% CO2 humidified incubator for 2–4 h. The absorbance value was read by a MRX II microplate reader (Dynex Technologies) at 440 nm and 650 nm (reference) and cell viability was counted. The experiment was repeated at least three times.

### Annexin-V/PI dual staining assay

Quantitative assessment of apoptosis was performed using a fluorescein isothiocyanate (FITC) Annexin V Apoptosis Detection Kit with Propidium Iodide (PI) (Biolegend). Briefly, cells were cultured (1 × 10^5^ cells) overnight prior to the treatment with SPHK1 inhibitors. Cells were then treated with SKI-II (5 μM), CAY10621 (10 μM) or Ceranib 2 (5 μM) for 48 h. Cells were washed once in PBS and resuspended in Annexin binding buffer and stained with FITC-conjugated Annexin V and PI for 15 min in the dark. The stained cells were diluted by the binding buffer and immediately analyzed by the flow cytometry (Becton Dickinson LSR II). Data acquired (1 × 10^5^ events per sample) was analyzed using the FlowJo software.

### Statistical analysis

Pearson’s Chi-squared test was used to test the association of the high SPHK1 expression with histological grade and other histopathological factors. The results for cell viability and apoptosis were presented as mean ± S. D, and statistical comparisons used two-tailed t-tests. In all cases, a *P* < 0.05 was considered statistically significant. All statistical analysis was performed by corresponding author.

## Additional files

Additional file 1: Histologic grading systems of feline mammary tumor used in this study. (JPG 85 kb)

Additional file 2: Representative images taken from tissues with various levels of SPHK1 expression. (PDF 148 kb)

Additional file 3: Box and whisker plot of the immunohistochemical intensity scores of SPHK1. (PDF 16 kb)

## Abbreviations

ER: Estrogen receptor
FMT: Feline mammary tumor
HER-2: Epidermal growth factor receptor 2
IHC: Immunohistochemical
S1P: Phingosine-1-phosphate
SPHK1: Sphingosine kinase 1
TMA: Tissue micro-array
