# Supplementary figures and images for: Significance of sphingosine kinase 1 expression in feline mammary tumors
Source: BMC Vet Res. 2019 May 17;15:155. doi: 10.1186/s12917-019-1883-z (PMC6525354; doi:10.1186/s12917-019-1883-z)

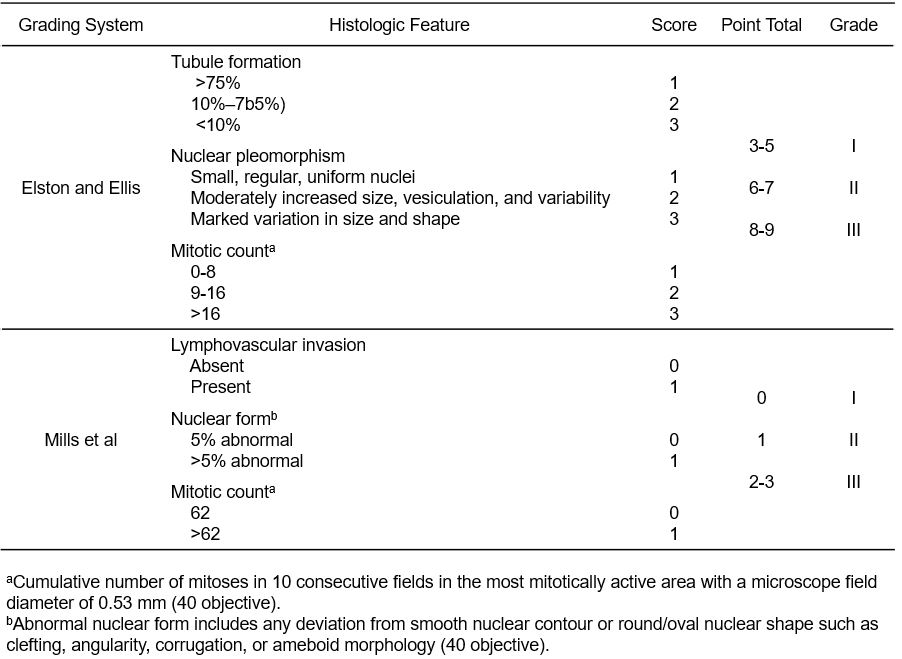

Supplement: Supplementary file 1 — Histologic grading systems of feline mammary tumor used in this study. (JPG 85 kb) [file 12917_2019_1883_MOESM1_ESM.jpg]

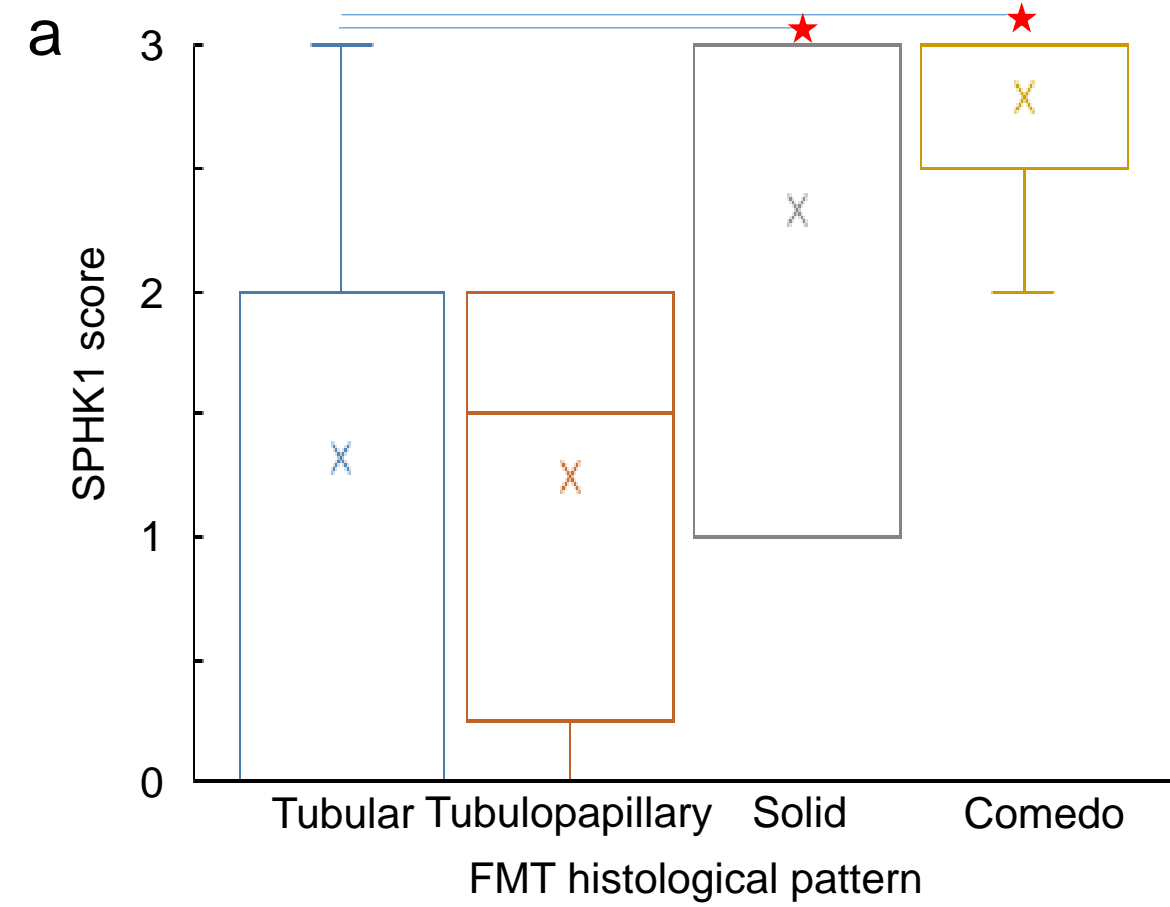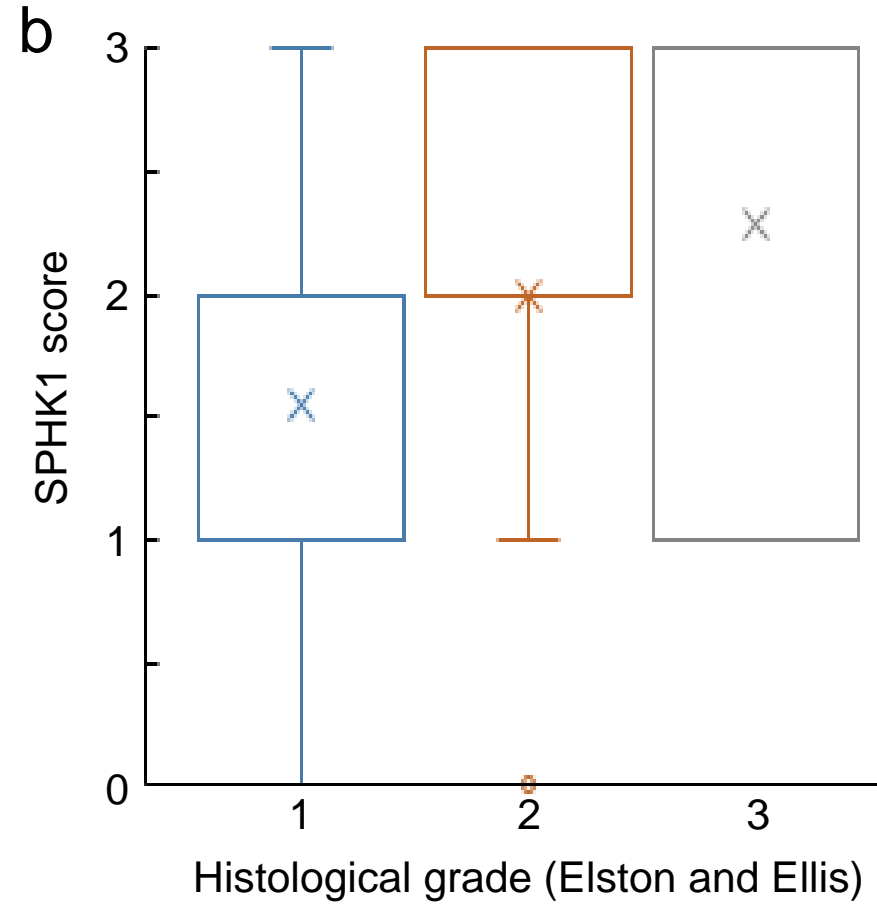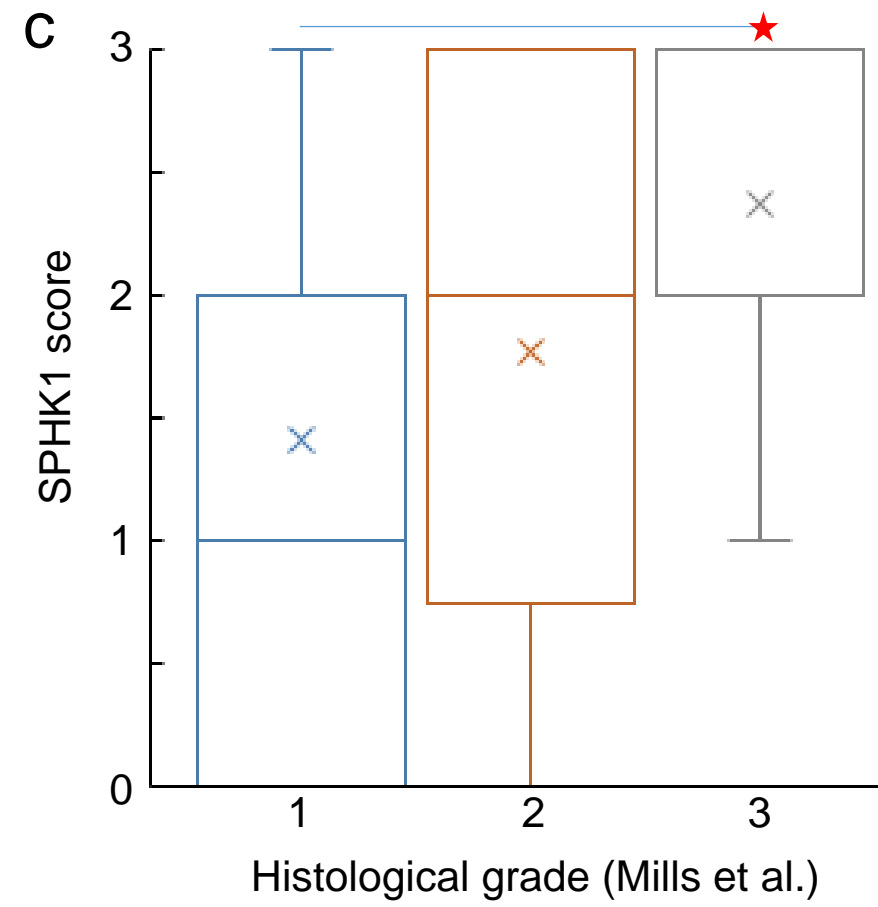

Supplement: Supplementary file 3 — Box and whisker plot of the immunohistochemical intensity scores of SPHK1. (PDF 16 kb) [file 12917_2019_1883_MOESM3_ESM.pdf]
