# Supplementary material for: Significance of sphingosine kinase 1 expression in feline mammary tumors
Source: BMC Vet Res. 2019 May 17;15:155. doi: 10.1186/s12917-019-1883-z (PMC6525354; doi:10.1186/s12917-019-1883-z)

Score	Staining pattern
0	No observable or weak staining
1+	Weak staining in malignant cells
2+	Moderate staining
3+	strong staining

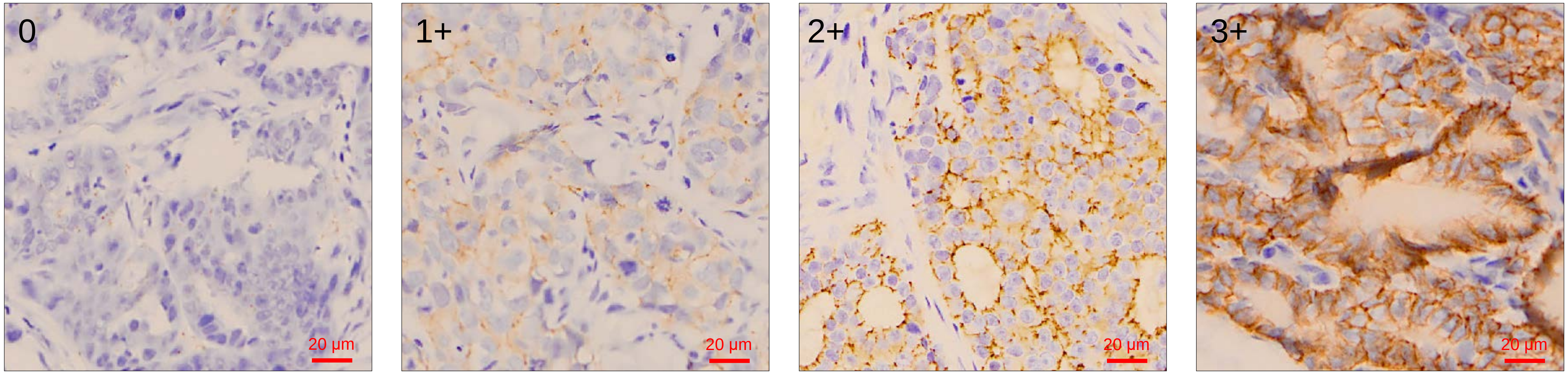

Supplement: Supplementary file 2 — Representative images taken from tissues with various levels of SPHK1 expression. (PDF 148 kb) [file 12917_2019_1883_MOESM2_ESM.pdf]
